# The Role of *MIR9-2* in Shared Susceptibility of Psychiatric Disorders during Childhood: A Population-Based Birth Cohort Study

**DOI:** 10.3390/genes10080626

**Published:** 2019-08-20

**Authors:** Luciana Tovo-Rodrigues, Gabriela Callo Quinte, Clarice Brinck Brum, Gabriele Ghisleni, Clarissa Ribeiro Bastos, Isabel Oliveira de Oliveira, Fernando C. Barros, Aluisio J. D. Barros, Iná S. Santos, Luis A. Rohde, Mara H. Hutz, Alicia Matijasevich

**Affiliations:** 1 Postgraduate Program In Health and Behavior, Catholic University of Pelotas., Pelotas, Rio Grande do Sul 96015-560, Brazil; 2 Laboratory of Clinical Neuroscience, Post-Graduate Program in Health and Behavior, Catholic University of Pelotas, Pelotas, Rio Grande do Sul 96015-560, Brazil; 3 Post-graduate Program Pediatrics Child Health, Pontifical Catholic University of Rio Grande do Sul, Porto Alegre, Rio Grande do Sul 90619-900, Brazil; 4 Department of Psychiatry, Hospital de Clínicas de Porto Alegre, Universidade Federal do Rio Grande do Sul, Porto Alegre, Rio Grande do Sul 90035-007, Brazil; 5 National Institute of Developmental Psychiatry for Children and Adolescents, São Paulo, São Paulo 05403-900, Brazil; 6 Program in Genetics and Molecular Biology Universidade Federal do Rio Grande Do Sul, Porto Alegre, Rio Grande do Sul 91501-970, Brazil; 7 Departamento de Medicina Preventiva, Faculdade de Medicina FMUSP, Universidade de São Paulo, São Paulo 01246-903, Brasil

**Keywords:** mental health, genetics, microRNA, birth cohort

## Abstract

Background: It has been suggested that microRNAs (miRNAs; short non-protein-coding RNA molecules that mediate post-transcriptional regulation), including mir-9 and mir-34 families, are important for brain development. Current data suggest that mir-9 and mir-34 may have shared effects across psychiatric disorders. This study aims to explore the role of genetic polymorphisms in the *MIR9-2* (rs4916723) and *MIR34B/C* (rs4938723) genes on the susceptibility of psychiatric disorders in children from the 2004 Pelotas Birth Cohort. Methods: Psychiatric disorders were assessed in 3585 individuals using Diagnostic and Statistical Manual of Mental Disorders, fourth edition (DSM-IV), criteria through the application of standard semi-structured interviews (using the Development and Well-Being Assessment, DAWBA) at the six-years-of-age follow-up. The outcome was defined as the presence of any mental disorder. We also considered two broad groups of internalizing and externalizing disorders to further investigate the role of these variants in mental health. Results: We observed an association between rs4916723 (*MIR9-2*) and the presence of any psychiatric disorder (odds ratios (OR) = 0.820; 95% CI = 0.7130–0.944; *p* = 0.006) and a suggestive effect on internalizing disorders (OR = 0.830; 95% CI = 0.698–0.987; *p* = 0.035). rs4938723 (*MIR34B/C*) was not associated with any evaluated outcome. Conclusion: The study suggests that *MIR9-2* may have an important role on a broad susceptibility for psychiatric disorders and may be important mainly for internalization problems.

## 1. Introduction

Psychiatric disorders with a childhood or adolescence onset are known to persist throughout life [1,2]. They are prevalent disorders with moderate to high genetic components, according to classic genetic evidences [3,4,5,6]. Shared symptoms and substantial epidemiological comorbidity incite debates about the extension of etiologic overlap. It has been shown that psychiatric disorders broadly share common genetic risk variants, mostly with neurodevelopmental effects [7], suggesting that current clinical boundaries do not reflect distinct underlying pathogenic processes, at least on the genetic level [7,8].

Dysregulation of complex gene networks in the developing brain is thought to underlie many of these disorders [9]. A growing body of evidence demonstrates that microRNAs (miRNAs), defined as short non-protein-coding RNA molecules that mediate post-transcriptional regulation by affecting miRNA stability and translation [10,11], play an important role on epigenetic regulation in brain functions and plasticity [12]. In fact, using the brain samples deposited on the Alen BrainSpan Atlas of the developing human brain, Ziats and Rennert (2014) [13] presented a spatio-temporal assessment of miRNA expression throughout early human brain development. The authors demonstrated that a set of miRNAs is differentially expressed along the brain development, suggesting that these molecules play critical roles in transcriptional networks of the developing human brain and neurodevelopmental disorders. miRNA-related genetic polymorphisms could potentially affect the maturation, expression, and structure of miRNAs, as well as their base pairing at the target site. It may, then, have a functional role in miRNA-mediated gene regulation, thereby affecting psychiatric diseases [14,15]. The role of miRNAs on psychiatric disorders has been explored in previous studies [16].

Among the miRNAs with described roles in the brain, mir-9 and mir-34 expressed transcript families are the most studied and have the greatest known relevance for brain processes. The gene *MIR9-2* is one of the genomic loci of the mir-9 family, whose relevance in neurogenesis has been extensively explored. Its role in the regulation of genes and pathways involved in differentiation of neural stem cells [17,18], proliferation and migration of neural progenitor cells [19], regulation of dopaminergic signaling [20], synaptic plasticity, and hippocampus-dependent memory [21] have been reported. In humans, mir-9 was observed to be differentially expressed in the hippocampus from infancy to early childhood [13], showing its importance for brain development processes. Alterations of the mir-9 expression in brain [22] and blood [23] tissues of patients with schizophrenia, in brain of Alzheimer’s disease patients [24], and the up-regulation in Parkinson’s disease treated patients compared to the untreated and the control groups [25] were observed, reinforcing its important role in the normal brain functions and development. In humans, *MIR9-2*, or its transcript expression, was already associated with schizophrenia [26], attention deficit and hyperactivity disorder (ADHD) [27], and neuroticism [28], and a pleiotropic effect across disorders has been reported [29]. rs4916723, placed about 108 kb downstream the *MIR9-2* gene, has already been associated with ADHD [27], with a set of psychiatric disorders [29], neuroticism [28], and alcohol consumption [30], and it was nominally associated with the autism spectrum disorder [31].

In mammals, the mir-34 family consists of three members, mir-34a, mir-34b, and mir-34c [32]. Together, the mir-34 family members are important regulators of neuronal development, plasticity, and disease. The proposed function of mir-34 family on neural stem cell differentiation, neurite elongation, neuronal differentiation, regulation of cell proliferation, and synaptogenesis has been reported [33,34]. rs4938723, placed in the *MIR34B/C* cluster promoter region, has been shown to modulate the expression of both genes [35]. It was already associated with major depression [36]. The mir-9 and mir-34 expression share a temporal profile, being differentially expressed in different brain regions from infancy (4 months–1 year) to early childhood (2–4 years) [13].

The elucidation of genetic influences and mechanisms shared across disorders may have an important impact on drug development, psychiatric nosology, and risk prediction. Considering the importance of miRNA-mediated gene regulation in brain development, it is expected that their effect emerges early in the susceptibility to psychiatric diseases. Although the relevance of mir-9 and mir-34 in several psychiatry disorders has been reported in the literature, to the best of our knowledge, their effects were not tested in populational-based studies where several disorders might be diagnosed. This study aims to explore the role of polymorphisms of the genes *MIR9-2* (rs4916723) and *MIR34B/C* (rs4938723) on the susceptibility of psychiatric disorders among children belonging to the 2004 Pelotas Birth Cohort study.

## 2. Methods

The present investigation uses data from the 2004 Pelotas birth cohort, a population-based study in the southern Brazilian city of Pelotas. Mothers of 4231 children accepted to participate in the study (refuses <1%) and were included in the study. So far, these children have been followed up at the ages of 3 and 12 months, 2, 4, 6, and 11 years, with follow-up rates of 95.7%, 94.3%, 93.5%, 92.0%, 90.2%, and 86.6%, respectively [37,38]. The present study used data from the perinatal assessment and the 6-year follow-up. At this follow-up (mean age: 6.6 years old, SD: 0.20), the children underwent a wide-range health assessment with interviews, including a saliva sample collection for DNA extraction [38]. The Oragene-250 kit model (DNA Genotek^®^, Ontario, Canada) was used for sample collection. DNA extraction was performed according to the manufacturer’s instructions. Only singleton children were included in this study.

### 2.1. Mental Health Outcomes

The psychiatric disorder diagnosis was obtained using the Development and Well-Being Assessment (DAWBA) [39], an instrument that consists of a structured part and open-ended questions about symptoms of psychiatric disorders, having questions based on Diagnostic and Statistical Manual of Mental Disorders, fourth edition (DSM-IV), diagnosis [40]. We used the Portuguese-language version of the DAWBA, which has been cross-culturally adapted and validated for use in Brazil [41]. The DAWBA was administered to mothers or caregivers by trained psychologists. More details can be found elsewhere [38,40].

At age 6, a total of 3585 (84.7% of 4231 births) children were assessed using DAWBA. Nearly 13% of the children presented any psychiatric disorder, according to DSM-IV. Anxiety disorders were the most prevalent (8.8%), followed by attention-deficit/hyperactivity disorders (ADHD, 2.6%), oppositional defiant disorder/conduct disorder (ODD/CD, 2.6%), and depression (1.3%). Autism (0.3%), Tic disorder and Tourette Syndrome (0.4%), eating disorders (0.03%), and stereotypies (0.1%) were also diagnosed [40].

We considered the presence of any of the mental disorders mentioned above as the main outcome in this study. We also aimed to explore the association between the polymorphisms and internalization/externalization disorders. We classified the disorders in two broad groups, as done by others [42]: Internalizing (comprised by any anxiety and depressive disorders) and externalizing (ADHD and ODD/CD) disorders.

### 2.2. Genetic Polymorphisms and Genotyping

Two polymorphisms were investigated: rs4916723, placed in chromosome 5, about 108 kb downstream to the *MIR9-2* gene (GRCh37 assembly), and rs4938723, placed in chromosome 11, about 1.1 kb and 1.6 kb upstream to *MIR34B/C* genes, respectively (GRCh37 assembly). Considering the relevance of the *MIR9-2* and *MIR34B/C* genes for brain development, the choice of polymorphisms around the genes was based on (1) results from studies about psychiatric disorders and related traits exploring the rs4916723 [27,28,29,30,31] and the rs4938723 [35,36], (2) minor allelic frequencies (MAF) in European and African continental populations higher than 0.10, and (3) technical viability (probes already designed and standardized by the manufacturer). The polymorphisms were genotyped using Taqman single nucleotide polymorphism (SNP) genotyping assays (Applied Biosystems, Foster City, CA, USA), according to the manufacturer’s recommended protocol for the allelic discrimination system (7500 Real Time PCR System, Applied Biosystems, Foster City, CA, USA).

### 2.3. Statistical Analyses

Allele and genotypic frequencies were estimated by counting. The Hardy–Weinberg equilibrium (HWE) was estimated through a chi-square test and implemented in the 1.9 PLINK software [43,44]. The threshold for the significance of HWE departure was set at 0.025, considering the adjustment for two tests (Bonferroni correction). In order to estimate the effect of individual markers, binary logistic regression was performed, using an additive genetic model and the major allele as the reference. The estimates are given through odds ratios (OR), 95% confidence intervals, and *p*-values. The analyses were performed using Stata v 14.0 (Stata LP, College Station, YX, USA).

For the association analysis, the alfa was corrected for multiple tests using the Bonferroni approach. The significant results were those with a *p* value lower than 0.008 (6 tests). Those tests comprising *p*-values between 0.008 and 0.05 were considered as suggestive associations. The adjusted regression model included skin color (white, brown, black, and others) and sex as confounders. As a sensitivity analysis, to minimize the possible effect of the environmental factors involving child-mother relationship, we run a model including maternal depression assessed by the Edinburgh Postnatal Depression Scale (EPDS) when the children were one year old. The datasets used and analyzed during the current study are available from the corresponding author on reasonable request.

### 2.4. In Silico Functionality Analysis

In order to provide further information about the putative regulatory function of each marker included in this study, we assessed HaploReg v4.1 [45]. It is a tool for exploring annotations of the noncoding genome in variants. Information on the chromatin state and protein binding annotation from the Roadmap Epigenomics and The Encyclopedia of DNA Elements (ENCODE) projects are functional evidences integrated in this dataset.

### 2.5. Ethics Approval and Consent to Participate

The School of Medicine Research Ethics Committee of the Federal University of Pelotas approved the study protocol and all follow-ups of the Pelotas 2004 Birth Cohort Study (ID 40870314.2.0000.5317). Informed consent was obtained from the guardians (mother, father, or legal guardian) of all participants, who signed the informed consent form.

## 3. Results

At the 6-year follow-up, a total of 3447 individuals had mental health information and valid genotypes for *rs4916723* and 3425 subjects for *rs4938723*. Amongst included participants, approximately 52% of the subjects were male, 68% had white skin color, and 43% of the mothers had studied for nine or more years. Table 1 shows the demographic and health characteristics of the probands included in the study and the genotypic frequency distribution for the two SNPs. Included and non-included participants had similar frequencies of boys and girls, skin color classification groups, and mother schooling. On the other hand, children included in the present analyses had lower frequencies of poorest and richest participants (according to the wealth index).

The minor allele frequencies were 0.368 (C-allele) for rs4916723 and 0.339 (C-allele) for rs4938723. No polymorphisms departed from the Hardy–Weinberg equilibrium (*p* = 0.15 for rs4916723, *p* = 0.04 for rs4938723).

Table 2 shows the association estimates for each polymorphism considering the presence of at least one psychiatric disorder considering DMS-IV diagnosis criteria for crude and adjusted models. The logistic regression using the additive model showed an association between rs4916723 (*MIR9-2*) and the presence of any psychiatric disorders after adjustment (odds ratios (OR) = 0.820; 95% CI = 0.713–0.944; *p* = 0.006). A protective effect of allele C was observed.

When the disorders were classified as internalizing or externalizing, a suggestive protective effect of allele C was observed for the internalizing group (OR = 0.830; 95% CI = 0.698–0.987; *p* = 0.035). For the externalizing group, the effect size was a little smaller and no association was observed. No association was observed for rs4938723 (*MIR34B/C*), both with the presence of any psychiatric disorder and for the test, including internalizing/externalizing subgroups. Similar results were found when we ran a model adjusting for maternal depression assessed in one year old children (Table S1).

In silico analysis was performed to explore the potential regulatory function of the investigated variants. The rs4916723 site is located in an intronic region of the *LINC00461* gene. According to HaploReg data, it is placed in a region of the promoter and enhances marks in the brain. It is also considered an expression quantitative trait loci (eQTL) for *LINC02060* in the same tissue (Table 3). Considering the regulatory regions specific in the brain, this SNP is placed in active domains of chromatin (Table 4), which are active sites (based on the Roadmap Epigenomics Consortium 15 chromatin state model) related to promoter and enhancer region for the fetal brain tissues, which is also confirmed by the DNAse hypersensitive sites. Moreover, the Roadmap Epigenomics Consortium 25 chromatin state model suggests that this SNP may play a role in adult brains as well (Table 4).

Considering the rs4938723 site, the elusive functional regulatory function in the brain was observed. However, histone marks were observed in other tissues (Table 3).

## 4. Discussion

In this study, we investigated the role of SNPs near the genes *MIR9-2* and *MIR34B/C* on mental health outcomes among children in a population-based Brazilian cohort. Our results suggest that rs4916723, close to the *MIR9-2*, has an important effect in the development of mental disorders, and more specifically in internalizing disorders. The findings are supported by the regulatory function of this SNP in the brain.

In this study, we observed an association between rs4916723 and the presence of any psychiatric disorders in childhood. Recent genome-wide association studies (GWAS studies) have indicated an important role of rs4916723 on several psychiatric diseases and related traits. A risk effect of the C-allele was already reported for ADHD in the most recent meta-analysis [27] as well as for several mental disorders (i.e., anorexia nervosa, ADHD, autism spectrum disorder, bipolar disorder, major depression, obsessive-compulsive disorder, schizophrenia, and Tourette syndrome) in a cross-psychiatric disorder meta-analysis of 232,964 cases and 494,162 controls [29]. A suggestive risk effect of the same allele was shown in the most recent autism spectrum disorder GWAS meta-analysis [31]. On the other hand, a protective effect of the same allele was already observed for alcohol consumption [30], neuroticism [28], which are phenotypes related to psychiatric disorders (Table S2). The latter are in agreement with the Brazilian findings. These findings reinforce its relevance in psychiatric disorders and they strengthen the need for further studies exploring the role of this marker on mental health. Differences in the mental disorders evaluated, age of assessment, and genetic background may explain the difference between the findings regarding the direction. Anxiety disorders was the most prevalent disorder in our study; however, they were not included in the published meta-analysis. Our sample included only children, while the meta-analysis included adults as well. The differences in the population background may also play a role on this divergence.

Our findings also suggest that the association considering rs4916723 may be specially related with internalizing disorders. Neuroticism, which may be defined as a tendency to experience negative emotions and high stress reactivity, has been pointed as an important factor for mood and anxiety, being suggested as the core feature for internalizing psychopathologies [46,47,48,49]. rs4916723 was also associated with neuroticism in a recent meta-analysis, in the same direction as our results, as mentioned above [28]. This role in neuroticism is in accordance with our suggestive association with internalizing disorders.

Although rs4916723 is close to *MIR9-2*, its function on the gene remains to be elucidated. This SNP is placed in the gene *LINC00461*, a non-coding RNA that is up-regulated in the brain [50], but without an established brain-specific role until the date. The *MIR9-2* gene is encoded on the same strand in the 3’ end of three splice forms of *LINC00461* [50], and the knockdown of *LINC00461* also drastically suppressed expression levels of mir-9, explicating the relation between both genes and providing the potential link between rs4916723 and *MIR9-2*. It is possible that this SNP is in linkage disequilibrium with other markers, leading to an indirect association with psychiatric disorders in our study. An important regulatory effect on the brain for rs4916723 is shown by HaploReg v.4.1 [45] data. The effect of this SNP in *MIR9-2* and the function of specific alleles on regulatory effects deserve further evaluation.

In our study, we did not observe a significant effect of *MIR34B/C* in the evaluated outcomes, contrasting with the results observed in ADHD [35] and depression [36] so far. rs4938723, in the *MIR34B/C* cluster promoter region, has been shown to modulate the expression of both genes and has an impact on the expression levels of genes regulated by both miRNA, including genes previously associated with ADHD and the central nervous system [38]. Mir-34b was observed to be differentially expressed in the dorsolateral prefrontal cortex and in the ventrolateral prefrontal cortex from infancy (4 months–1 year) to early childhood (2–4 years) [13]. Mir-34c was also differentially expressed in the dorsolateral prefrontal cortex from infancy to early childhood. The association considering this polymorphism should be addressed in future studies.

Our results must be interpreted in the context of some limitations. There were small differences between the extreme socioeconomic groups of the included and not included participants. However, the response rate was high, which helps attenuate possible selection bias. Regarding the externalizing/internalizing tests, it is important to note that the size effect for the externalizing group was a little smaller, with a wide confidence interval. Although an important effect on internalizing disorders was observed, we cannot exclude the hypothesis that this polymorphism may also play a role on externalizing. Further studies should address this topic. On the other hand, our findings were identified from a majority-white population, and our results may not be generalizable to populations with other ethnic backgrounds. Although results from the city of Pelotas do not indicate population stratification in the region [51] and the allelic effect is similar in white and non-white individuals in our samples, we are not able to rule out populational stratification potential bias, since we do not have the population genomic. Moreover, given the small prevalence of psychiatric disorders at the age of six, it was not possible to consider every single diagnosed disorder, which would benefit the disease-specificity effect exploration of the SNPs included in this study.

## 5. Conclusions

Finally, to the best of our knowledge, this is the first study exploring the role of *MIR9*-2 and the *MIR43B/C* cluster on mental health susceptibility in a large population-based cohort. The study suggests that *MIR9-2* may have an important role on a broad susceptibility for psychiatric disorders, mainly those related with internalization. It also shows the need for other studies to better understand the extension of shared genetic effects in psychiatric diseases.

## Figures and Tables

**Table 1:** Socio demographic characteristics and genotype frequencies distribution among the participants of the 2004 Pelotas birth cohort.

Variables	Included (*n* = 3447)	Not included (*n* = 784)	*p*-Value
Sex	*n* (%)	*n* (%)	
Male	1780 (51.6)	415 (52.9)	0.513
Female	1667 (48.4)	369 (47.1)	
Skin color			0.137
White	2332 (67.7)	394 (71.4)	
Black	428 (12.4)	54 (9.8)	
Others	686 (19.9)	104 (18.8)	
Mother schooling (y)			0.057
<5	512 (15.0)	142 (18.4)	
5–8	1426 (41.7)	305 (39.6)	
>9	1478 (43.3)	323 (42.0)	
Wealth Index (quintiles)			0.001
1st (poorest)	579 (20.9)	128 (25.6)	
2nd	523 (18.9)	97 (19.4)	
3rd	551 (19.9)	84 (16.8)	
4th	576 (20.8)	74 (14.8)	
5th (richest)	536 (19.5)	117 (23.4)	
Genotypes *			
rs4916723			
AA	1356 (39.3)	-	
AC	1644 (47.7)	-	
CC	447 (13.0)	-	
rs4938723			
TT	1522 (44.5)	-	
CT	1482 (43.3)	-	
CC	420 (12.2)	-	

* rs4916723 Hardy–Weinberg equilibrium (HWE): *p*-value: Chi-square = 2.18, *p* = 0.140; rs4938723 chi-square = 4.03, *p*-value: 0.045.

**Table 2:** Association analysis between single nucleotide polymorphisms (SNPs) and mental health outcomes at six years of age using logistic regression (crude and adjusted models) and an additive genetic model.

Outcome	*n*	rs4916723 (*MIR9-2*) OR_crude_ (95% CI)	rs4916723 (*MIR9-2*) * OR_adj_ (95% CI)	* *p_adj_*	*N*	rs4938723 (*MIR34B/C*) OR_crude_ (95% CI)	rs4938723 (*MIR34B/C*) * OR_adj_ (95% CI)	* *p_adj_*
			Effect Allele: C			Effect Allele: C		
Any disorder	**3447**	**0.816 (0.710–0.938)**	**0.820 (0.713–0.944)**	**0.006**	3424	1.047 (0.915–1.198)	1.055 (0.921–1.208)	0.442
Any externalizing disorder	3447	0.863 (0.672–1.108)	0.870 (0.676–1.118)	0.276	3424	1.063 (0.835–1.353)	1.074 (0.843–1.368)	0.566
Any internalizing disorder	**3447**	**0.824 (0.693–0.979**)	**0.830 (0.698–0.987)**	**0.035**	3424	0.994 (0.841–1.176)	1.002 (0.847–1.185)	0.984

* Regression model adjusted by skin color and sex. Sample size post adjustment: 3446 for rs4916723 and 3424 for rs4938723. N = Sample size included in the crude models. Internalizing disorders comprise any depressive and anxiety disorders. Externalizing disorders comprise attention deficit and hyperactivity disorder (ADHD), oppositional defiant disorder (ODD) and conduct disorder (CD). The significant and suggestive results are denoted in bold.

**Table 3:** Single nucleotide polymorphism (SNP) regulatory information available in Haploreg v4.1.

SNP ID	CHR	Position (GRCh37)	Gene/Location	Promoter Histone Marks Tissues	Enhancer Histone Marks Tissues	DNAse Tissues	Motifs Changed	eQTL Hits
rs4916723	5	87854395	*LINC00461* intronic	BRN	BRN, ESC, iPSC, BRN.CRTX	BRN	11 altered motifs	*LINC02060* in Brain Hippocampus and Nucleus accumbens
rs4938723	11	111382565	*BTG4* intronic	ESC, BRST	ESC, iPSC, FAT.ADIP, SKIN.PEN.FRSK.,BRST, SKIN.NHEK	iPSC, BRST	3 altered motifs	*COLCA2* in Colon Transverse PPP2R1B in Whole Blood

Chromosome (CHR); Expression quantitative trait loci (eQTL); brain (BRN); embryonic stem cells (ESC); induced pluripotent stem cells (iPSC); adipose-derived mesenchymal stem cell cultured cells (FAT.ADIP); foreskin fibroblast primary cells (SKIN); foreskin melanocyte primary cells (SKIN.PEN.FRSK); Breast (BRST) epidermal Keratinocyte Primary Cells (SKIN.NHEK); Cortex-derived primary cultured neurospheres (BRN.CRTX). Long intergenic non-protein coding RNA 461 (LINC00461); BTG anti-proliferation factor 4 (BTG4); long intergenic non-protein coding RNA 2060 (LINC02060); colorectal cancer associated 2 (COLCA2); protein phosphatase 2 scaffold subunit Abeta (PPP2R1B): Search conducted on 18 May 2019.

**Table 4:** Regulatory sites in brain regions available for the two evaluated SNPs (HaploReg v4.1).

Epigenome ID (EID)	Group	Description	Chromatin states (Core 15-State Model)	Chromatin States (25-State Model Using 12 Imputed Marks)	H3K4me1	H3K4me3	H3K27ac	H3K9ac	DNase
rs4916723									
E071	Brain	Brain Hippocampus Middle	-	22_PromP	H3K4me1_Enh	H3K4me3_Pro	-	-	-
E074	Brain	Brain Substantia Nigra	-	-	H3K4me1_Enh	H3K4me3_Pro	-	-	-
E068	Brain	Brain Anterior Caudate	-	22_PromP	H3K4me1_Enh	H3K4me3_Pro	-	H3K9ac_Pro	-
E069	Brain	Brain Cingulate Gyrus	-	17_EnhW2	--	-	-	-	-
E072	Brain	Brain Inferior Temporal Lobe	-	-	H3K4me1_Enh	-	-	-	-
E067	Brain	Brain Angular Gyrus	-	19_DNase	H3K4me1_Enh	H3K4me3_Pro	-	-	-
E073	Brain	Brain_Dorsolateral_Prefrontal_Cortex	-	17_EnhW2		H3K4me3_Pro	H3K27ac_Enh	H3K9ac_Pro	-
E070	Brain	Brain Germinal Matrix	2_TssAFlnk	16_EnhW1	H3K4me1_Enh	H3K4me3_Pro	-	-	-
E082	Brain	Fetal Brain Female	2_TssAFlnk	13_EnhA1	H3K4me1_Enh	H3K4me3_Pro	-	-	DNase
E081	Brain	Fetal Brain Male	7_Enh	16_EnhW1	H3K4me1_Enh	-	-	-	DNase
rs4938723									
E071	Brain	Brain Hippocampus Middle	-	-	-	H3K4me3_Pro	H3K27ac_Enh	-	-
E074	Brain	Brain Substantia Nigra	-	-	-	-	H3K27ac_Enh	H3K9ac_Pro	-
E068	Brain	Brain Anterior Caudate	-	-	-	H3K4me3_Pro	H3K27ac_Enh	H3K9ac_Pro	-
E069	Brain	Brain Cingulate Gyrus	-	-	H3K4me1_Enh	H3K4me3_Pro	H3K27ac_Enh	-	-
E072	Brain	Brain Inferior Temporal Lobe	-	-	-	H3K4me3_Pro	-	H3K9ac_Pro	-
E067	Brain	Brain Angular Gyrus	-	-	H3K4me1_Enh	H3K4me3_Pro	-	H3K9ac_Pro	-
E073	Brain	Brain_Dorsolateral_Prefrontal_Cortex	-	-	-	-	-	-	-
E070	Brain	Brain Germinal Matrix	-	-	-	-	-	-	-
E082	Brain	Fetal Brain Female	-	-	-	-	-	-	-
E081	Brain	Fetal Brain Male	-	-	-	-	-	-	-

## Supplementary Materials

The following are available online at https://www.mdpi.com/2073-4425/10/8/626/s1, Table S1: Association analysis between SNPs and mental health outcomes at 6 years of age using logistic regression (crude and adjusted models) and additive genetic model. The adjusted model includes the correction for maternal depression. Table S2: Summary of recent GWAS findings regarding rs4916723 on psychiatric disorders and related traits.
