# Supplementary material for: The Role of MIR9-2 in Shared Susceptibility of Psychiatric Disorders during Childhood: A Population-Based Birth Cohort Study
Source: Genes (Basel). 2019 Aug 20;10(8):626. doi: 10.3390/genes10080626 (PMC6723948; doi:10.3390/genes10080626)
Supplement: Supplementary file 1 [file genes-10-00626-s001.pdf]

genes

MDPI

Supplementary Material

Table S1. Association analysis between SNPs and mental health outcomes at 6 years of age using logistic regression (crude and adjusted models) and additive genetic model. The adjusted model includes the correction for maternal depression.

**Outcome**	*n*	**rs4916723 (*MIR9-2*) OR$_{crude}$ (95% CI)**	**rs4916723 (*MIR9-2*) *OR$_{adj}$ (95% CI)**	*p_{adj}	*N*	**rs4938723 (*MIR34B/C*) OR$_{crude}$ (95% CI)**	**rs4938723 (*MIR34B/C*) *OR$_{adj}$ (95% CI)**	*p_{adj}
		Effect Allele: C				Effect Allele: C		
Any disorder by DMS-IV	**3447**	**0.816 (0.710–0.938)**	**0.811 (0.701–0.940)**	**0.005**	3424	1.047 (0.915–1.198)	1.044 (0.906–1.203)	0.555
Any externalizing disorder	3447	0.863 (0.672–1.108)	0.923 (0.676–1.118)	0.54	3424	1.063 (0.835–1.353)	1.138 (0.843–1.368)	0.313
Any internalizing disorder	**3447**	**0.824 (0.693–0.979)**	**0.834 (0.698–0.997)**	**0.04**	3424	0.994 (0.841–1.176)	1.000 (0.837–1.183)	0.955

* Regression model adjusted by skin color, sex and maternal depression assessed when the children were 1 year old. Sample size post adjustment: 3324 for rs4916723 and 3301 for rs4938723. N= Sample size included in the crude models. Internalizing disorders comprise any depressive and anxiety disorders. Externalizing disorders comprise ADHD, oppositional defiant disorder and conduct disorder. The significant and suggestive results are denoted in bold.

Table S2. Summary of recent GWAS findings regarding rs4916723 on psychiatric disorders and related traits.

**Study**	**Phenotype**	**Allele**	**Effect size**	***p*-value**	**Retrieved from**	**Sample size**	**Population**
Grove et al. (2019)	ASD	A	0.964 (Odds Ratio)	1.924×10^{-6}	Summary statistics File: iPSYCH-PGC_ASD_Nov2017	18,382 cases and 27,969 controls	iPSYCH-PGC European ancestry
Demontis et al. (2019)	ADHD	A	0.926 (Odds Ratio)	1.58×10^{-8}	Top hit reported in the main paper	20,183 cases, 35,191 controls	iPSYCH-PGC, All ancestries
Lee et al (2019)	Several psychiatric diseases (Cross-trait GWAS meta-analysis)	A	NA	1.82×10^{-9}	Top hit reported in the main paper	232,964 cases and 494,162 controls	* European Ancestry
Nagel et al. (2018)	Neuroticism	A	5.609 (z-score)	2.03×10^{-8}	Supplementary Table 2	434,007	UKB/23andMe/GPC1 European ancestry
Liu et al. (2019)	Alcohol consumption (drinks per week)	C	-1.15×10^{-2} (beta)	8.07×10^{-9}	Summary Statistics File: DrinksPerWeek.txt	537,349	GSCAN cohorts European ancestry

*AN (Anorexia; Duncan et al., 2017), ASD (Autism Spectrum Disorder; Grove et al., 2017), ADHD (Attention Deficit/Hyperactivity Disorder; Demontis et al., 2019), BIP (Bipolar Disorder; Stahl et al., .2018), MD (Major Depression; Wray et al., 2018), OCD (Obsessive Compulsive Disorder; International Obsessive Compulsive Disorder Foundation Genetics Collaborative (IOCDF-GC) and OCD Collaborative Genetics Association Studies (OCGAS), 2018), TS (Tourette Syndrome; Yu et al., In press.), and

SCZ (Schizophrenia; Schizophrenia Working Group of the Psychiatric Genomics, 2014). GSCAN: GWAS and Sequencing Consortium of Alcohol and Nicotine use; UKB: UK Biobank; GPC1: Genetics of Personality Consortium; PGC: Psychiatric Genomics Consortium; NA: Not available

© 2019 by the authors. Submitted for possible open access publication under the terms and conditions of the Creative Commons Attribution (CC BY) license (http://creativecommons.org/licenses/by/4.0/).
